# Effect of metformin on anti-mullerian hormone levels in women with polycystic ovarian syndrome: a systematic review and meta-regression analysis of randomized controlled trials with

**DOI:** 10.1186/s12902-024-01570-z

**Published:** 2024-03-29

**Authors:** Mehdi Mehdinezhad Roshan, Mohammad Hassan Sohouli, Elma Izze da Silva Magalhães, Azita Hekmatdoost

**Affiliations:** 1 Student Research Committee, Department of Biology and Anatomical Sciences, School of Medicine, Shahid Beheshti University of Medical Sciences, Tehran, Iran; 2 Student Research Committee, Department of Clinical Nutrition and Dietetics, Faculty of Nutrition and Food Technology, Shahid Beheshti University of Medical Sciences, Tehran, Iran; 3 Federal University of Rio Grande do Sul, Postgraduate Program in Food, Nutrition and Health, Porto Alegre, Rio Grande do Sul, Brazil; 4 Department of Clinical Nutrition and Dietetics, Faculty of Nutrition and Food Technology, Shahid Beheshti University of Medical Sciences, Tehran, Iran

**Keywords:** Metformin, AMH, Women, PCOS, Meta-analysis

## Abstract

**Background:**

Several interventional studies have evaluated the potential anti-Mullerian hormone (AMH)-reduction effect of metformin. However, the results are still contradictory. In order to obtain a better viewpoint from them, this study aimed to comprehensively investigate the effects of metformin on AMH in the women with with polycystic ovarian syndrome (PCOS).

**Methods:**

Scopus, PubMed/Medline, Web of Science, Cochrane, and Embase databases were searched using standard keywords to identify all controlled trials investigating the AMH levels following metformin administration. Pooled weighted mean difference and 95% confidence intervals were achieved by random-effects model analysis for the best estimation of outcomes.

**Results:**

Sixteen studies with 484 participants’ were included in this article. The pooled findings showed that AMH levels in the single arm clinical trials were significantly reduced (pooled WMD of -3.06 ng/ml; 95% confidence interval [CI] -4.03 to -2.10; *P* < 0.001) after use of metformin. Furthermore, compared to the control group, in randomized clinical trials, a reduced significant effect on AMH levels was observed following use of metformin (pooled WMD of -3.47 ng/ml; 95% CI -7.14 to -0.19; *P* = 0.047). Furthermore, higher reduction in the AMH levels with a metformin dosage ≤ 1500 mg/day and duration of treatment ≤ 12 weeks when compared to higher dosages and duration of intervention, observed in this meta-analysis.

**Conclusions:**

In conclusion, results this meta-analysis of clinical trials confirms the beneficial effect of the treatment with metformin in the reduction of the AMH levels in women.

**Supplementary Information:**

The online version contains supplementary material available at 10.1186/s12902-024-01570-z.

## Introduction

Anti-Mullerian hormone (AMH) is a dimeric glycoprotein member of the superfamily of peptide growth/differentiation factors as such the transforming growth factor-β (TGF-β) [1]. In women, AMH is expressed by the granulosa cells and secreted into the circulation from birth to menopause. At birth, levels of AMH are hard to detectable, however, it increases significantly at puberty, slowly reducing during the reproductive period, until it become undetectable at menopause [2].

In addition to being an ovarian reserve marker, AMH plays a potential role in the pathophysiology diagnosis and treatment of several ovarian pathologies, including polycystic ovary syndrome (PCOS) [2, 3]. In PCOS, AMH levels are higher due to an increase in the number of antral follicles and in the production pre antral follicle. Although not yet accepted as a diagnostic criterion for PCOS, AMH is very useful in anticipating hyperresponsiveness to ovarian stimulation. AMH neutralizes the action of follicle-stimulating hormone (FSH) and thus may play a role in PCOS ovulatory disorders [4]. Moreover, in PCOS, increased levels of circulating insulin contribute to hyperandrogenism, which causes a derangement in folliculogenesis, thereby contributing to polycystic ovary morphogenesis and higher than normal AMH [5, 6].

Due to the role of the insulin resistance in the pathophysiology of PCOS and increased of the AMH levels, insulin-sensitizing drugs as such metformin have been used in the treatment of this syndrome, having been postulated that such agents would reduce the insulin resistance and, consequently, AMH levels in women with PCOS [5, 7].

Yin et al. [8] performed a meta-analysis to assess the effect of using seven types of drugs, including metformin, on AMH levels in women of reproductive age, which showed a significant reduction in AMH levels in women with PCOS after treatment with metformin. However, we identified additional studies with potential for inclusion in a new meta-analysis, which may increase the robustness and accuracy of the analyses. Furthermore, to our knowledge, to date, no systematic review and meta-analysis including meta-regression analysis has been published on the topic.

To conduct a systematic meta-analysis investigating the influence of metformin on anti-Mullerian hormone (AMH) levels in women, utilizing data from 16 relevant studies with a total of 484 participants. The primary goal is to assess the overall impact of metformin on AMH levels, considering both single-arm and randomized clinical trials. Additionally, the objective includes examining the dosage and duration-dependent effects of metformin treatment on AMH reduction, with a specific focus on lower dosages (≤ 1500 mg/day) and shorter durations (≤ 12 weeks). The study aims to provide insights into the potential benefits of metformin in lowering AMH levels among women and encourages further research and individualized clinical considerations to enhance the understanding of these observed effects.

## Methods

### Search strategy

The Preferred Reporting Items for Systematic Review and Meta-analysis (PRISMA) criteria were followed for conducting this study [9]. Without regard to language or time restrictions, a thorough search was carried out in the PubMed/MEDLINE, Web of Science, SCOPUS, and Embase databases from the beginning to March 2023. Additionally, similar papers and gray literature were considered in the search. Medical subject headings (MeSH) and Emtree (Embase subject headings) were selected to search the online databases, as follow: (“Metformin” OR “Dimethylbiguanidine” OR “Glucophage” OR “Metformin HCl” OR “Metformin Hydrochloride”) AND (“Anti-Mullerian Hormone” OR “AMH” OR " Mullerian Inhibiting Factor” OR “Mullerian Inhibitory Substance” OR ” Mullerian Inhibiting Hormone” OR ” Mullerian Regression Factor”) AND (“Clinical Trials as Topic” OR “Cross-Over Studies” OR “Double-Blind Method” OR “Single-Blind Method” OR “Random Allocation” OR “Clinical Trial”). The reference lists of the publications retrieved and linked review studies were manually searched to identify potentially overlooked qualifying trials.

### Eligibility criteria

Using titles, abstracts, or the complete texts of the research, two writers separately removed duplicate articles before finding and reviewing relevant publications. In the end, the papers were separated based on the following standards: (1) Randomized or single arm clinical trials studies; (2) metformin has been given as an intervention in individual’s aged 12 and over with PCOS (PCOS, was diagnosed based on the Rotterdam consensus statements criteria, and included at least two of the following three characteristics: menstrual irregularity due to oligo and/or anovulation (having an interval of > 35 days between menstrual periods and/or amenorrhea, described as the absence of vaginal bleeding for at least six months), polycystic ovaries morphology on ultrasound exam (12 or more small follicles in an ovary), and clinical symptoms or biochemical markers of hyperandrogenism and exclusion of other etiologies [10]); and (3) baseline and post in both group (intervention and control) AMH was recorded. The most recent or longest follow-up period was used when a research revealed results at more than one follow-up time. Studies with duplicated data, studies with ambiguous information, studies in which metformin was used as an intervention alongside other commonly prescribed medications, non-single arm or randomized trial designs, animal studies, reviews, and meta-analysis studies were also excluded.

### Data extraction

The qualifying studies were examined by two authors independently. The first author’s name, the study’s location, the year it was published, the sample size (for the intervention and control groups), the participant characteristics (such as the percentage of men, the participant’s BMI, age, and health status), the type of outcomes, duration of the intervention, the dosage and type of the intervention, and the means and standard deviations (S.D.s) of the intended outcomes at baseline, post-intervention, and/or changes between baseline and post-intervention, were all extracted.

### Quality assessment

The details of the evaluation of the study’s quality are presented in Table 1. Using the Cochrane risk-of-bias test for randomized trials (RoB 2), version 2, the quality of the included RCTs was methodologically evaluated [11]. Based on the following potential sources of bias: blinding of outcome assessment, allocation concealment, participant and staff blinding, random sequence generation, incomplete outcome data, selective reporting, and other bias, two authors independently rated each study as having a low, high, or unclear risk of bias. Any discrepancies were discussed with a third author in order to come to a consensus. The NutriGrade (Grading of Recommendations Assessment, Development, and Evaluation) grading method was also used to evaluate the quality of the current analytic research [12]. A reliable 10-point assessment system that assesses elements affecting study quality is the NutriGrade checklist. This scale has seven components: (1) risk of bias, (2) precision, (3) heterogeneity, (4) directness, (5) publishing bias, (6) funding bias, and (7) study design.

**Table 1:** Risk of bias assessment according to the Cochrane collaboration’s risk of bias assessment tool

Study, Year (reference)	Random sequence generation	Allocation concealment	Blinding of participants and personnel	Blinding of outcome assessment	Incomplete outcome data	Selective reporting	Overall assessment of risk of bias
*Foroozanfard et al.*	Unclear	Unclear	Unclear	Unclear	Low	Low	Unclear
*Tomova et al.*	Low	Unclear	Unclear	Low	Low	Low	Unclear
*Sauerbrun-Cutler et al.*	Unclear	Unclear	Unclear	Unclear	Unclear	Unclear	Unclear
*Nascimento et al.*	Low	Unclear	Unclear	Unclear	Low	Low	Unclear
*Panidis et al.*	Low	Unclear	Low	Low	Low	Unclear	Unclear
*Neagu et al.*	Low	Low	Unclear	Low	Low	Low	Unclear
*Romualdi et al.*	Unclear	Unclear	Unclear	Unclear	Low	Low	Unclear
*Piltonen et al.*	Unclear	Unclear	Unclear	Unclear	Unclear	Unclear	Unclear
*Dursun et al.*	Low	Low	Unclear	Low	Unclear	Low	Unclear
*Fleming et al.*	Unclear	Unclear	Unclear	Unclear	Unclear	Unclear	Unclear
*Falbo et al.*	Unclear	Unclear	Unclear	Unclear	Unclear	Unclear	Unclear
*Wiweko et al.*	High	High	Unclear	Unclear	Unclear	Low	High
*Tagliaferri et al.*	Low	Low	Unclear	Low	Low	Low	Unclear
*Chhabra et al.*	Unclear	Unclear	Unclear	Unclear	Low	Low	Unclear
*Madsen et al.*	Low	Unclear	Unclear	Unclear	Low	Low	Unclear
*Fabregues et al.*	Unclear	Unclear	Unclear	Unclear	Unclear	Unclear	Unclear

### Data synthesis and statistical analysis

The data were examined using STATA version 12.0 software. Different data types were converted using a predetermined procedure to the mean and standard deviations (S.D.s) [13, 14]. For instance, in the absence of standard deviations, we calculated the change using the method below: The definition of standard deviation changes is square root [(S.D. baseline 2 + SD final 2) - (2R S.D. baseline 2 S.D. final)]. The following formula is used to convert the standard error of the mean (SEM) to standard deviation: S.D. is equal to SEM × √n, where n is the total number of participants in each group. The random-effects model was employed in the meta-analysis of research results. The weighting of the research followed the typical inverse variance technique. The data from the longest time point were used for the analysis, which allowed for the handling of many assessments within a single study group. Using Q Statistics and I-squared (I2), the degree of study heterogeneity was evaluated. Insignificant, low, moderate, and high heterogeneity were found with I2 values ranging from 0% to 25, 26–50%, 5–75%, and 76–100%, respectively [15]. Meta-regression investigates whether particular covariates (potential effect modifiers) explain any of the heterogeneity of treatment effects between studies. Thus, meta-regression between metformin and absolute mean differences in AMH level based on dosage of and duration of intervention was performed using random effect model. To identify possible causes of heterogeneity, a pre-defined subgroup analysis based on the dosage and duration of the intervention were conducted. A sensitivity analysis was done to determine the contribution of each research to the overall mean difference. In order to establish if there was publication bias, we utilized the official Egger’s test [16].

## Results

**Fig. 1:**
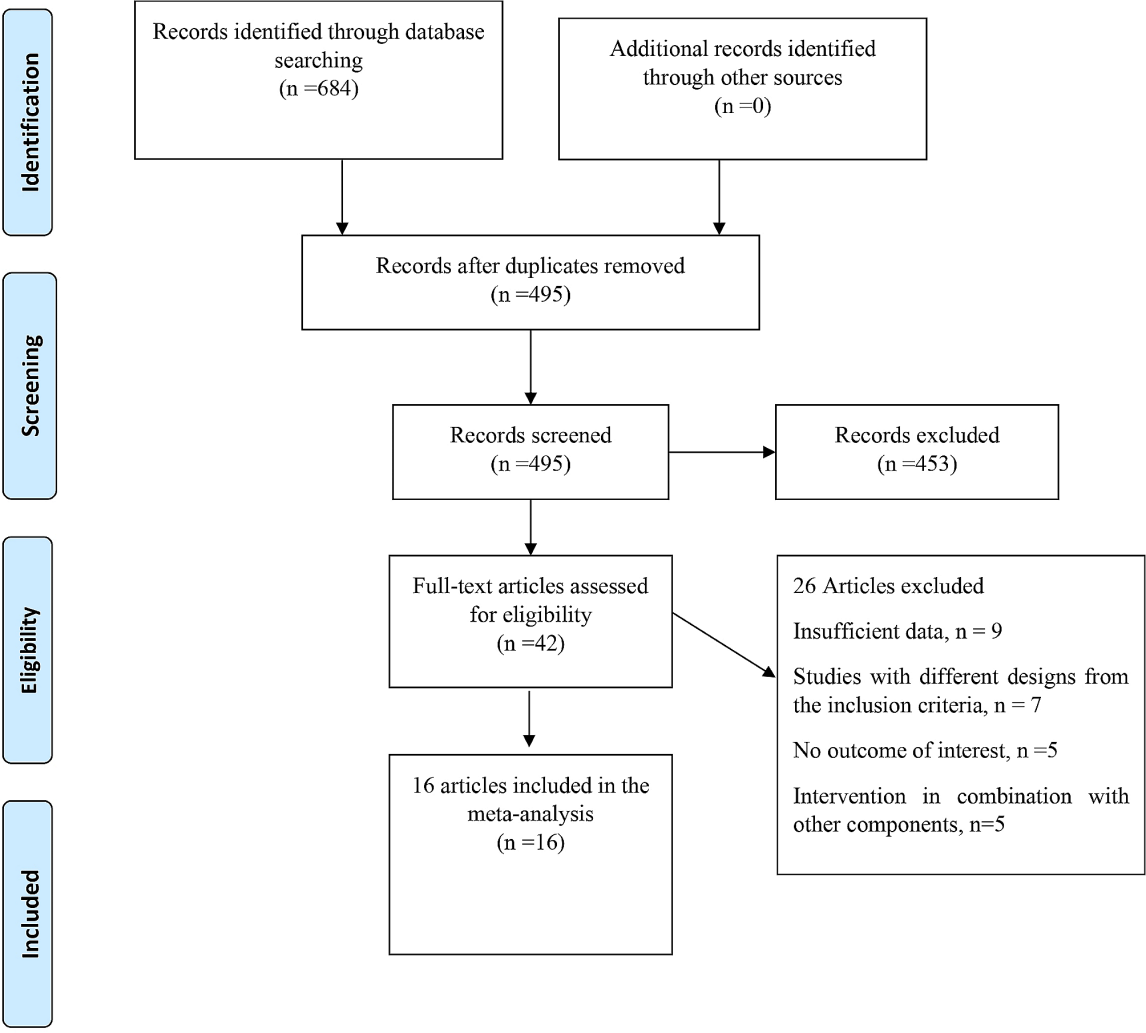
Flow chart of the included studies, including identification, screening, eligibility and the final sample included

Figure 1 depicts a flowchart of the research selection process with exclusion criteria. This value indicates that the aforementioned electronic databases generated 684 articles. After removing publications with duplicate research, there were 495 total. Following an assessment of the research’s titles and abstracts, 453 papers were dropped since they didn’t meet the inclusion requirements. 42 articles were found utilizing the full-text search during the secondary screening. For the reasons listed above, 26 of the investigations were dropped. Finally, 16 [5, 17–31] papers with 19 treatments arm were included in the quantitative meta-analysis since they matched the qualifying requirements.

### Study characteristics

The features of the pooled articles are shown in Table 2. Our surveys reveal that three studies were conducted in the American continent, 2 studies in the Asian continent and the rest of the articles in the European continent. All articles were published between 2005 and 2018 and follow up intervention ranged from 8 to 48 weeks. The mean age of participants ranged from 15.2 to 32.06 years at the baseline. The doses prescribed in the studies were between 1500 and 2550 mg per day, and 11 studies were conducted with single arm clinical trial design and 5 studies were conducted as RCT. The mean BMI at the baseline level was between 21.56 and 37.1 in the studies included. In addition, all the participants in the studies were PCOS patients.

**Table 2:** Characteristics of eligible studies

Author	Publication Year	Country	Study Design	Mean Age year	Sample Size intervention	Sample Size control	Duration of intervention (Weeks)	Dose of intervention (mg/day)	Type of control	Mean BMI	
Foroozanfard et al.	2017	Iran	Single arm clinical trial	25.3	30	-	8	1500	-	26.1	
*Tomova et al.*	*2011*	*Bulgaria*	*Single arm clinical trial*	*26.59*	*13*	*-*	*24*	*2550*	*-*	*30.73*	
*Sauerbrun-Cutler et al.*	*2012*	*USA*	*Single arm clinical trial*	*31.5*	*16*	*-*	*12*	*1500*	*-*	*NR*	
*Nascimento et al.*	*2013*	*Brazil*	*Single arm clinical trial*	*26.3*	*16*	*-*	*8*	*1500*	*-*	*29.1*	
*Panidis et al.*	*2010*	*Greece*	*Single arm clinical trial*	*21.07*	*15*	*-*	*24*	*1700*	*-*	*21.56*	
*Neagu et al.*	*2012*	*Romania*	*Single arm clinical trial*	*NR*	*11*	*-*	*8*	*2550*	*-*	*NR*	
*Romualdi et al.*	*2011*	*Italy*	*Single arm clinical trial*	*24.4*	*28*	*-*	*24*	*1700*	*-*	*33.59*	
*Piltonen et al.*	*2005*	*Finland*	*Single arm clinical trial*	*30.6*	*26*	*-*	*24*	*1500*	*-*	*30.1*	
*Dursun et al.*	*2015*	*Turkey*	*Single arm clinical trial*	*15.2*	*20*	*-*	*24*	*2000*	*-*	*30.6*	
*Fleming et al.*	*2005*	*USA*	*Single arm clinical trial*	*30.2*	*82*	*-*	*32*	*1500/2550*	*-*	*37.1*	
*Falbo et al.*	*2010*	*Italy*	*Single arm clinical trial*	*27.4*	*10*	*-*	*48*	*1700*	*-*	*23*	
*Wiweko et al.*	*2017*	*Indonesia*	*Randomized clinical trial (parallel)*	*28.25*	*20*	*18*	*48*	*1500*	*100 mg DLBS3233 DLBS3233 is a herbal medicine produced in Indonesia*	*28.02*	
*Tagliaferri et al.*	*2017*	*Italy*	*Randomized clinical trial (crossover)*	*25.62*	*34*	*34*	*24*	*1700*	*Placebo*	*32.55*	
*Chhabra et al.*	*2018*	*India*	*Randomized clinical trial (parallel)*	*28.81*	*35*	*35*	*24*	*1700*	*Placebo*	*NR*	
*Madsen et al.*	*2015*	*Denmark*	*Randomized clinical trial (crossover)*	*31.5*	*45*	*42*	*12*	*1700*	*Placebo*	*33.7*	
*Fabregues et al.*	*2010*	*Spain*	*Randomized clinical trial (parallel)*	*32.06*	*15*	*15*	*24*	*1700*	*Placebo*	*25.6*	

BMI: body mass index; N/R: not reported

The findings of the evaluation of the eligible studies’ quality are shown in Table 1. Additionally, a score of 7.7 (good quality) was determined after the NutriGrade score system was used to assess the quality of the current meta-analysis.

### Meta-analysis results

**Fig. 2:**
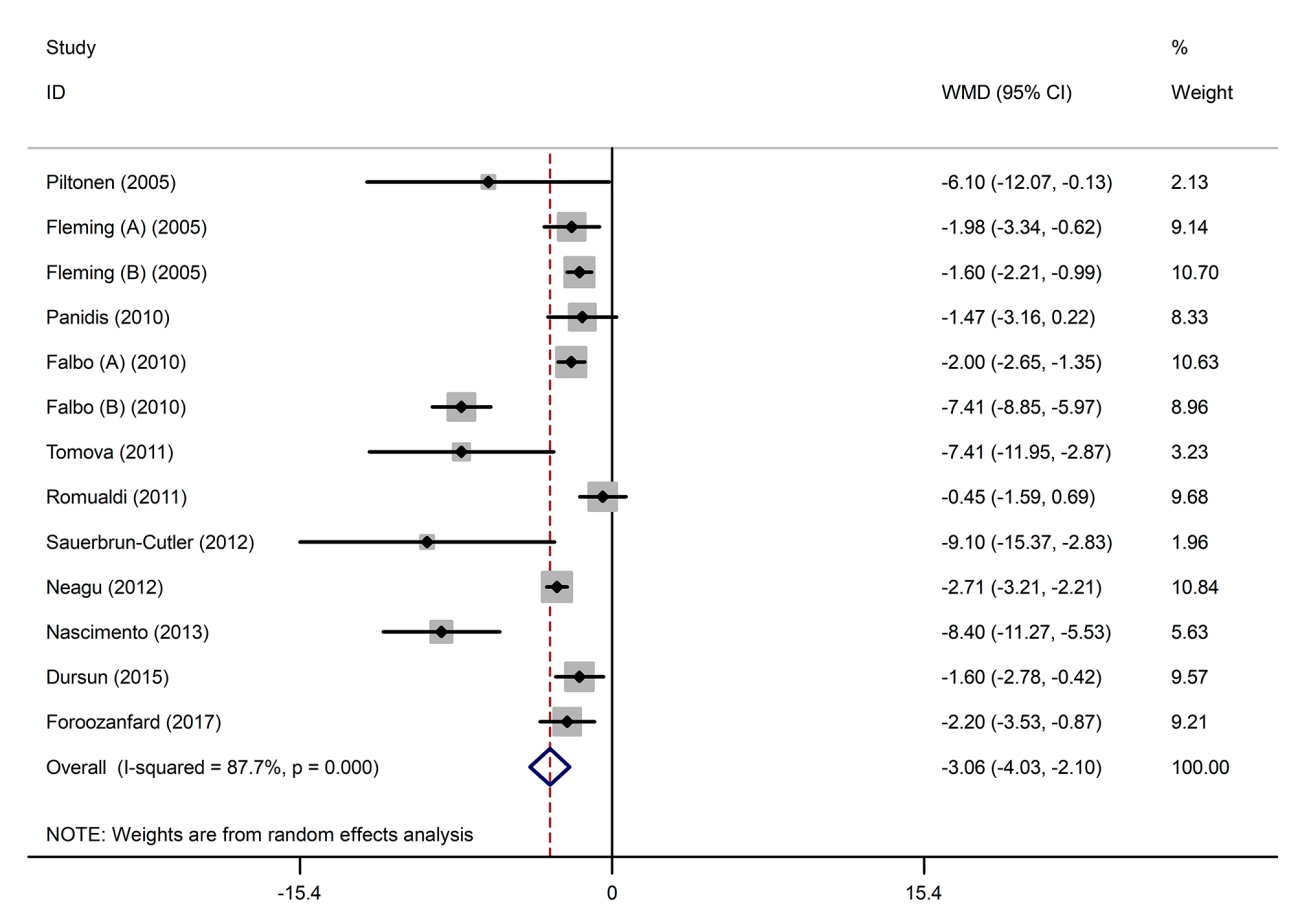
Forest plot of single arms clinical trial of the effects of metformin on anti-mullerian hormone (AMH) (ng/ml) levels

**Fig. 3:**
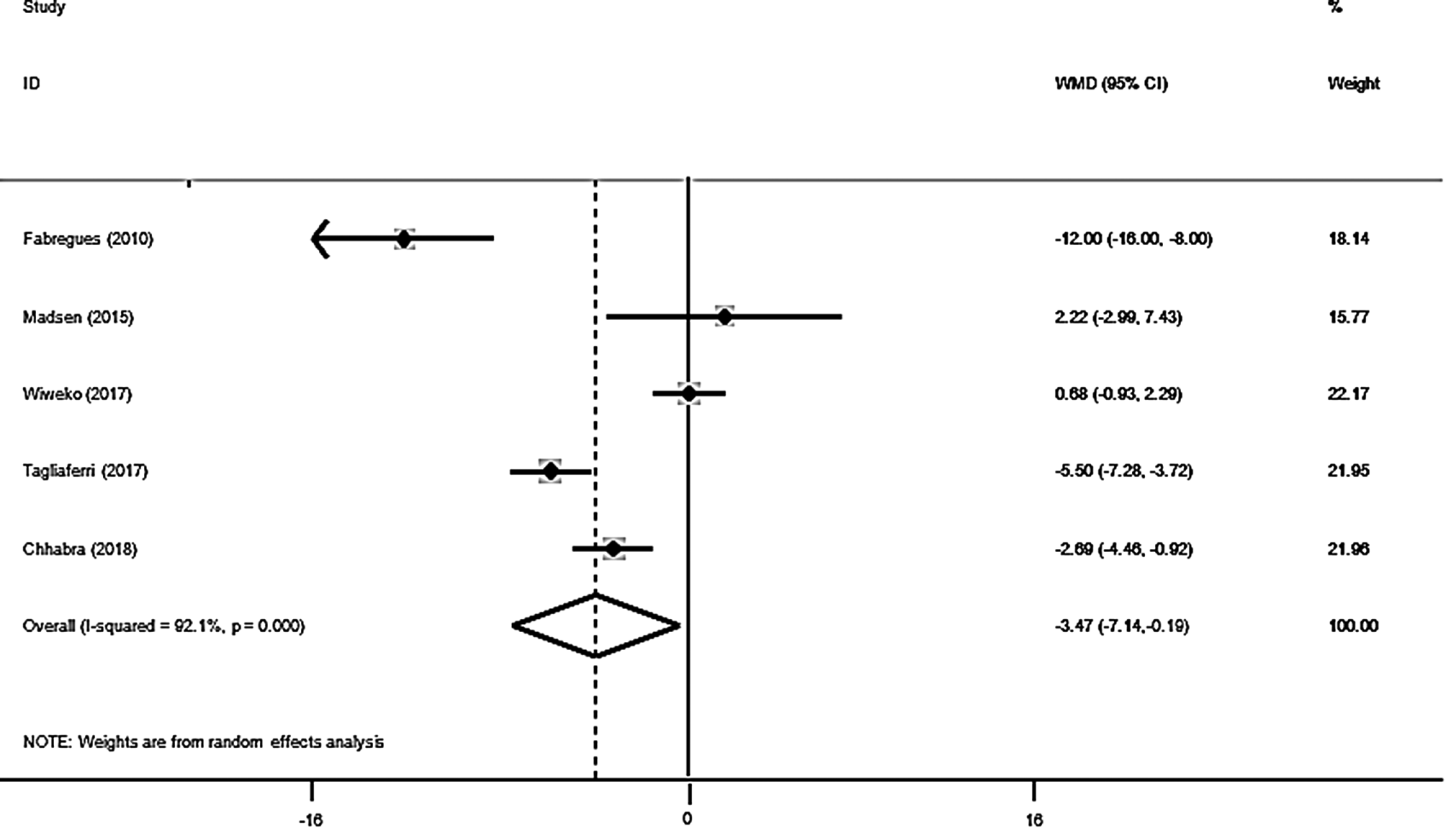
Forest plot of randomized controlled trials investigating the effects of Metformin on Anti-Mullerian Hormone (AMH) (ng/ml) levels

Pooled findings of the meta-analysis of random-effects model indicated that AMH levels in the single arm clinical trials were significantly reduced (pooled WMD of -3.06 ng/ml; 95% confidence interval [CI] -4.03 to -2.10; *P* < 0.001) after use of metformin. Furthermore, compared to the control group, in randomized clinical trials, a reduced significant effect on AMH levels was observed following use of metformin (pooled WMD of -3.47 ng/ml; 95% CI -7.14 to -0.19; *P* = 0.047). Also, significant heterogeneity was noted among the studies for this outcome (Cochran *Q* test, *P* < 0.001, I^2^ = 87.7% for single arms clinical trials and Cochran *Q* test, *P* < 0.001, I^2^ = 92.1% for randomized clinical trials). Subgroup analysis were performed to find the possible origin of heterogeneity **(**Figs. 2 and 3**)**.

### Subgroup analysis

**Fig. 4:**
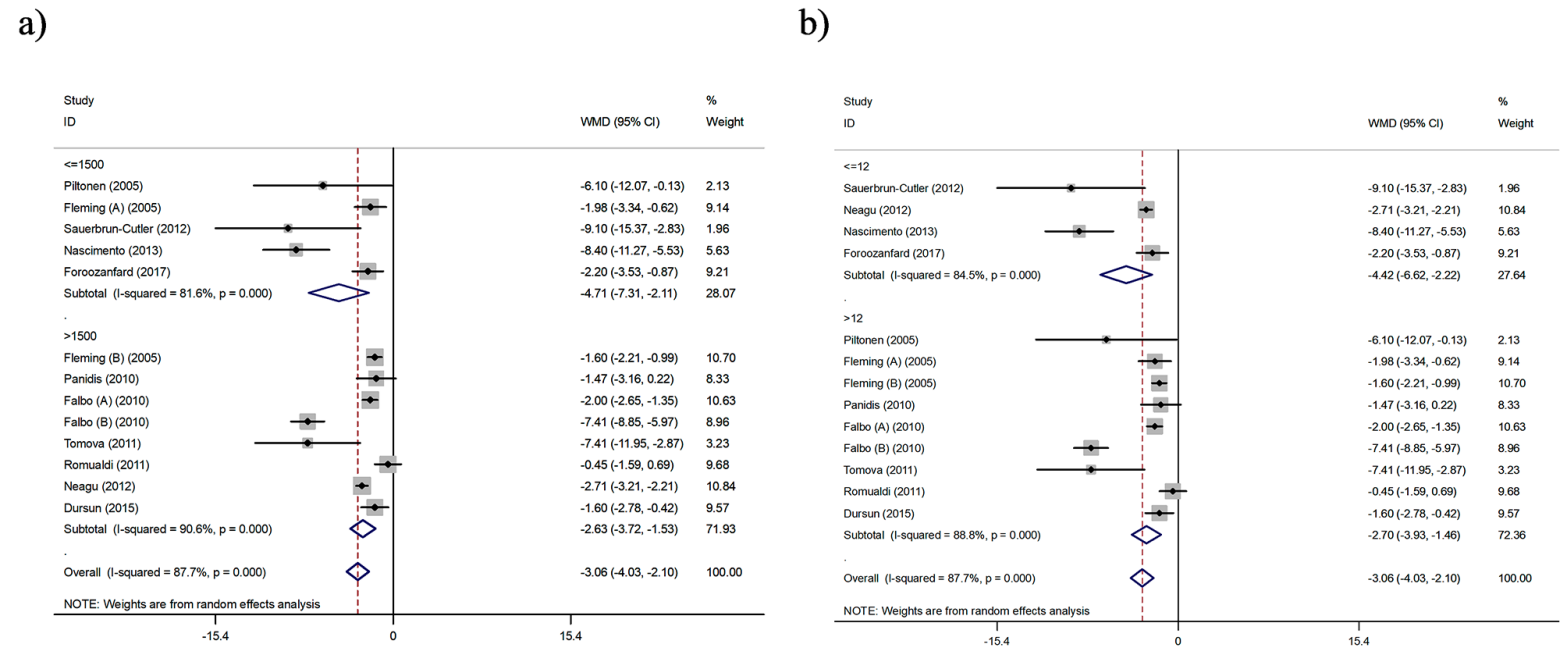
Forest plot of single arms clinical trial of the effects of Metformin on Anti-Mullerian Hormone (AMH) (ng/ml) levels based on (**a**) dose of intervention (mg) and (**b**) duration of study (week)

We subsequently stratified the articles based on the dosage of metformin (mg) and duration of intervention (weeks) in the single arms clinical trial studies **(**Fig. 4**)**. The results of subgroup analysis showed that metformin during the intervention ≤ 12 weeks (WMD: -4.42 ng/ml, 95% CI: -6.62 to -2.22, I^2^ = 84.5%) and with a dose ≤ 1500 mg per day (WMD: -4.71 ng/ml, 95% CI: -7.31 to -2.11, I^2^ = 81.6%) causes a greater decrease in AMH levels than the intervention > 12 weeks (WMD: -2.70 ng/ml, 95% CI: -3.93 to -1.46, I^2^ = 88.8%) and at a dose > 1500 mg (WMD: -2.63 ng/ml, 95% CI: -3.72 to -1.53, I^2^ = 90.6%).

### Meta-regression

**Fig. 5:**
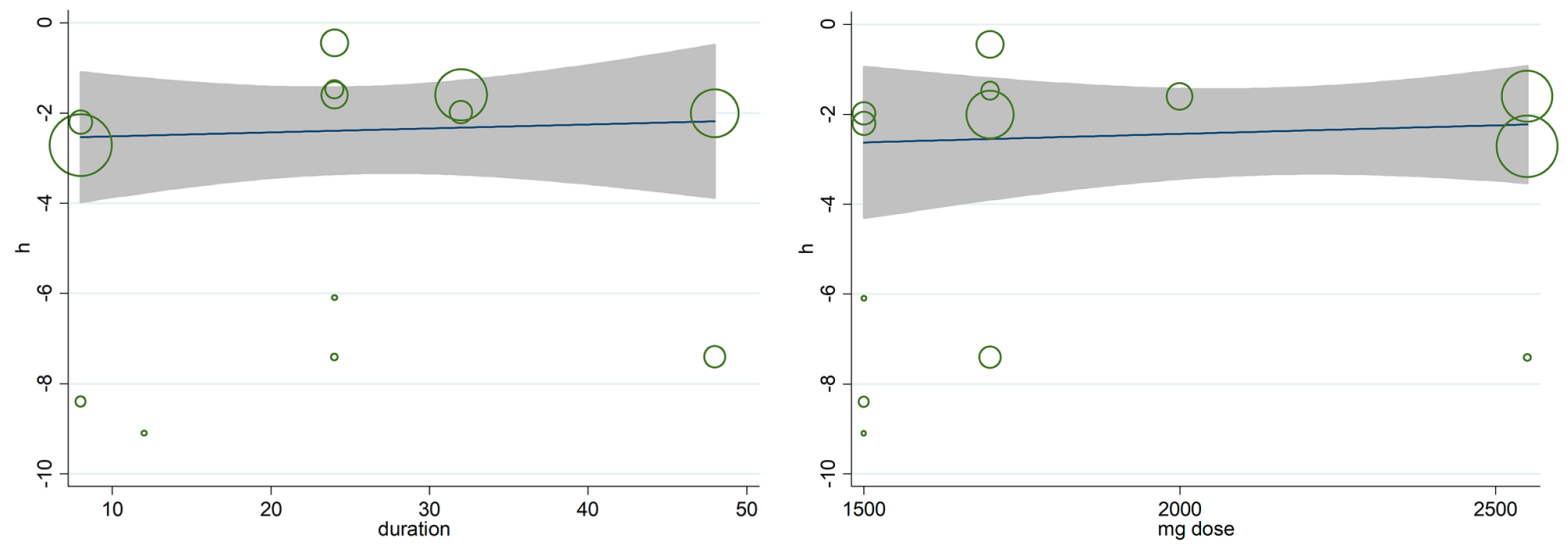
Meta-regression analysis encompassing AMH changes according to the duration of intervention (Weeks), dose of intervention (mg) in the single arms clinical trial

Meta-regression to estimate the AMH levels regarding on absolute dosage and duration of the metformin use were performed, but no significance was observed (Coef = 0.0020715, *P* = 0.446 for dose of intervention; Coef = 0.0437079, *P* = 0.490 for duration of intervention; see Fig. 5).

### Sensitivity analysis

In order to discover the effect of each article (in the single arm and randomized clinical trials) on the pooled effect size for AMH levels, we step-by-step discarded each trial from the analysis. The leave-one-out sensitivity analysis indicated the robustness of the results (Supplementary Fig. 1).

### Publication bias

Evaluating the publication bias by visual inspection of the funnel, no evidence for publication bias based on the Begg’s tests were detected (*P* = 0.071 for single arms clinical trial studies for *P* = 0.327 for randomized clinical trial studies) (Supplementary Fig. 2).

## Discussion

Results of meta-analyses of random-effects models indicated for both single arm clinical trials and randomized clinical trials that the use of metformin reduced AMH levels. Besides, in single-arm clinical trials, a higher reduction in the AMH levels was observed with metformin dosage ≤ 1500 mg per day and duration of treatment ≤ 12 weeks. However, in the meta-regression analysis, no significant results were observed for AMH levels in relation to absolute dosage and duration of metformin use.

Results from a previous meta-analysis, which included 12 clinical trials, showed a 1.79 ng/ml reduction (WMD: -1.79, 95%CI: -2.32 to -1.26) in AMH levels after metformin treatment in women with PCOS [8]. On the other hand, by including more studies, our meta-analysis showed even greater significant reductions in AMH levels after the use of metformin in women with PCOS, both in single-arm clinical trials and in randomized controlled clinical trials.

Metformin is the most common antihyperglycemic agent, being also indicated for treatment of patients with PCOS. As a first-line treatment for insulin resistance, metformin can improve insulin sensitivity and control blood glucose levels, which can also reduce androgen levels and improve ovulation [7, 32]. In this sense, AMH levels are a useful biochemical marker of prognostic for metformin treatment in PCOS [7].

Higher reduction in the AMH levels with a metformin dosage ≤ 1500 mg/day and duration of treatment ≤ 12 weeks when compared to higher dosages and duration of intervention, observed in this meta-analysis, suggests that a dosage of until 1500 mg metformin daily for until three months would already be effective enough for considerable reduction of AMH levels in women with PCOS. It is important to highlight that treatment with metformin at higher doses and for a prolonged period is associated with gastrointestinal alterations [33], reinforcing that it is valid to seek the lowest effective dose and the shortest time of use to reduce adverse effects to patients. The results of subgroup analyses according to treatment dosage or duration are, however, surprising. We hypothesize that individuals with polycystic ovary syndrome (PCOS) who are prescribed greater doses of metformin or longer treatment durations may experience a more severe disease state, such as severe insulin resistance. Increasing the dosage or extending the therapy of metformin is unlikely to change its impact on AMH levels. The decrease in AMH levels due to metformin medication may not be readily apparent in these individuals. However, it is possible that excessive doses and/or prolonged use of metformin could have side effects that hinder its effectiveness in normalizing AMH levels. These subgroup analyses suggest that the severity of disease in PCOS patients can predict the effectiveness of metformin treatment.

No significant results in the meta-regression analysis, in turn, indicates that the relation of the absolute dosage and use duration of metformin with AMH levels in patients with PCOS is not linear, suggesting that there is not a biological gradient in this relation.

Women with PCOS have AMH serum levels that are 2–3 times greater than those of healthy women [34, 35]. Granulosa cells of pre-antral and small antral follicles mostly release this hormone. Due to enhanced specific secretion and increased follicle numbers, there may be more of this hormone in the blood than usual [34, 36]. Insulin stimulates the activation of primordial follicles, according to research conducted on animals [37]. Pituitary gonadotropins are partially required for the activation of primordial follicles, according to investigations on hypophysectomized animals [38]. Another prevalent component that may be linked to PCOS and contribute to anovulation, hyperandrogenism, and a rise in AMH in this condition is hyperinsulinemia [39]. AMH inhibits the activation of primordial follicles [40]. Thus, metformin and other insulin-sensitizing medications are utilized by the majority of PCOS-afflicted females [29].

The current study has some strengths despite its limitations, including a rigorous methodology based on the PRISMA guidelines, a thorough literature search that included multiple independent databases, separate and duplicate searches, selection of the selected studies, and data extraction, as well as the use of a third party to resolve disagreements. Additionally, the current study probably had the biggest impact size for each outcome evaluated at PCOS.

The following are limitations. First off, the accuracy of the subgroup analysis of GnRH-a may have been compromised because we were unable to get in touch with several authors to gather the original data. Second, even though we were able to find pertinent publications in several databases, there are certain unpublished data that we are still unable to access. Thirdly, it is challenging to control the confounding factors because original research included a variety of control groups, including healthy women, infertile women, elderly women, and women with varied PCOS diagnostic criteria. Other limitations of this study include the low quality of reviewed articles, lack of examination of subtypes of PCOS, as well as some confounding factors affecting the disease, such as lifestyle and use of oral contraceptives.

In conclusion, results this meta-analysis of clinical trials confirms the beneficial effect of the treatment with metformin in the reduction of the AMH levels in women, presumably by an insulin-dependent mechanism of action, which may be useful in the treatment and control of the PCOS. For clinical practice, the findings suggest that the combination of lower daily doses of metformin (up to 1500 mg) for a shorter period (up to 3 months) is already sufficient to verify the effects of this drug on AMH levels with a better gastrointestinal tolerability, thus this dosage and duration should be preferred when treating patients with PCOS.

### Electronic supplementary material

Below is the link to the electronic supplementary material.

Supplementary Material 1

## Data Availability

Data is available upon request from the corresponding author for the article due to privacy / ethical restrictions.

## References

[1] Grootegoed JA, Baarends WM, Themmen AP (1994). Welcome to the family: the anti-müllerian hormone receptor. Mol Cell Endocrinol.

[2] Grynnerup AG, Lindhard A, Sørensen S (2012). The role of anti-Müllerian hormone in female fertility and infertility - an overview. Acta Obstet Gynecol Scand.

[3] Dumont A, Robin G, Catteau-Jonard S, Dewailly D (2015). Role of Anti-Müllerian hormone in pathophysiology, diagnosis and treatment of polycystic ovary syndrome: a review. Reprod Biol Endocrinol.

[4] Bhide P, Homburg R (2016). Anti-Müllerian hormone and polycystic ovary syndrome. Best Pract Res Clin Obstet Gynaecol.

[5] Chhabra N, Malik S. Effect of insulin sensitizers on raised serum anti-mullerian hormone levels in Infertile women with polycystic ovarian syndrome. J Hum Reprod Sci. 2018 Oct-Dec;11(4):348–52. PubMed PMID: 30787519. Pubmed Central PMCID: PMC6333035. eng. 10.4103/jhrs.JHRS_59_17 PMC6333035 30787519

[6] Speroff L, Fritz MA. Clinical gynecologic endocrinology and infertility. lippincott Williams & wilkins; 2005.

[7] Dunaif A (2008). Drug insight: insulin-sensitizing drugs in the treatment of polycystic ovary syndrome—a reappraisal. Nat Clin Pract Endocrinol Metab.

[8] Yin W-W, Huang C-C, Chen Y-R, Yu D-Q, Jin M, Feng C (2022). The effect of medication on serum anti-müllerian hormone (AMH) levels in women of reproductive age: a meta-analysis. BMC Endocr Disorders.

[9] Moher D, Shamseer L, Clarke M, Ghersi D, Liberati A, Petticrew M (2015). Preferred reporting items for systematic review and meta-analysis protocols (PRISMA-P) 2015 statement. Syst Reviews.

[10] Adams J, Polson D, Franks S (1986). Prevalence of polycystic ovaries in women with anovulation and idiopathic hirsutism. Br Med J (Clin Res Ed).

[11] Higgins JP, Savović J, Page MJ, Elbers RG, Sterne JA. Assessing risk of bias in a randomized trial. Cochrane Handb Syst Reviews Interventions. 2019;205:28.

[12] Schwingshackl L, Knüppel S, Schwedhelm C, Hoffmann G, Missbach B, Stelmach-Mardas M (2016). Perspective: NutriGrade: a Scoring System to assess and judge the Meta-evidence of Randomized controlled trials and Cohort studies in Nutrition Research. Adv Nutr.

[13] Higgins J. Cochrane handbook for systematic reviews of interventions. Version 5.1. 0 [updated March 2011]. The Cochrane Collaboration. www.cochrane-handbook.org. 2011.

[14] Hozo SP, Djulbegovic B, Hozo I (2005). Estimating the mean and variance from the median, range, and the size of a sample. BMC Med Res Methodol.

[15] Higgins JP, Thompson SG, Deeks JJ, Altman DG (2003). Measuring inconsistency in meta-analyses. BMJ.

[16] Egger M, Smith GD, Schneider M, Minder C (1997). Bias in meta-analysis detected by a simple, graphical test. BMJ.

[17] Wiweko B, Susanto CA (2017). The effect of metformin and cinnamon on serum anti-mullerian hormone in women having PCOS: a Double-blind, randomized, controlled trial. J Hum Reproductive Sci.

[18] Foroozanfard F, Samimi M, Almadani KH, Sehat M (2017). Effect of metformin on the anti-Müllerian hormone level in infertile women with polycystic ovarian syndrome. Electron Physician.

[19] Tagliaferri V, Romualdi D, Immediata V, De Cicco S, Di Florio C, Lanzone A (2017). Metformin vs myoinositol: which is better in obese polycystic ovary syndrome patients? A randomized controlled crossover study. Clin Endocrinol.

[20] Tomova А, Deepinder F, Robeva R, Kirilov G, Mechandjiev Z, Kumanov P (2011). Anti-Müllerian hormone in women with polycystic ovary syndrome before and after therapy with metformin. Horm Metab Res.

[21] Sauerbrun-Cutler M, Lederman M, Keltz M, Lee M, Stein D (2012). Serum anti-müllerian hormone levels (AMH) decrease after metformin administration in women with both lean and overweight polycystic ovary syndrome (PCOS). Fertil Steril.

[22] Nascimento AD, Lara LAS, Rosa-e-Silva ACJS, Ferriani RA, Reis RM (2013). Effects of metformin on serum insulin and anti-mullerian hormone levels and on hyperandrogenism in patients with polycystic ovary syndrome. Gynecol Endocrinol.

[23] Panidis D, Georgopoulos NA, Piouka A, Katsikis I, Saltamavros AD, Decavalas G (2011). The impact of oral contraceptives and metformin on anti-Müllerian hormone serum levels in women with polycystic ovary syndrome and biochemical hyperandrogenemia. Gynecol Endocrinol.

[24] Neagu M, Cristescu C (2012). Anti-Műllerian hormone–a prognostic marker for metformin therapy efficiency in the treatment of women with infertility and polycystic ovary syndrome. J Med Life.

[25] Romualdi D, De Cicco S, Tagliaferri V, Proto C, Lanzone A, Guido M (2011). The metabolic status modulates the effect of metformin on the antimullerian hormone-androgens-insulin interplay in obese women with polycystic ovary syndrome. J Clin Endocrinol Metabolism.

[26] Piltonen T, Morin-Papunen L, Koivunen R, Perheentupa A, Ruokonen A, Tapanainen JS (2005). Serum anti-Müllerian hormone levels remain high until late reproductive age and decrease during metformin therapy in women with polycystic ovary syndrome. Hum Reprod.

[27] Dursun F, Güven A, Yıldız M (2016). Assessment of anti-Müllerian hormone level in management of adolescents with polycystic ovary syndrome. J Clin Res Pediatr Endocrinol.

[28] Madsen HN, Lauszus FF, Trolle B, Ingerslev HJ, Tørring N (2015). Impact of metformin on anti-Müllerian hormone in women with polycystic ovary syndrome: a secondary analysis of a randomized controlled trial. Acta Obstet Gynecol Scand.

[29] Fleming R, Harborne L, MacLaughlin DT, Ling D, Norman J, Sattar N (2005). Metformin reduces serum müllerian-inhibiting substance levels in women with polycystic ovary syndrome after protracted treatment. Fertil Steril.

[30] Fábregues F, Castelo-Branco C, Carmona F, Guimerá M, Casamitjana R, Balasch J (2011). The effect of different hormone therapies on anti-müllerian hormone serum levels in anovulatory women of reproductive age. Gynecol Endocrinol.

[31] Falbo A, Rocca M, Russo T, D’Ettore A, Tolino A, Zullo F (2010). Serum and follicular anti-mullerian hormone levels in women with polycystic ovary syndrome (PCOS) under metformin. J Ovarian Res.

[32] Campagnoli C, Berrino F, Venturelli E, Abbà C, Biglia N, Brucato T (2013). Metformin decreases circulating androgen and estrogen levels in nondiabetic women with breast cancer. Clin Breast Cancer.

[33] Blonde L, Dailey GE, Jabbour SA, Reasner CA, Mills DJ (2004). Gastrointestinal tolerability of extended-release metformin tablets compared to immediate-release metformin tablets: results of a retrospective cohort study. Curr Med Res Opin.

[34] Pigny P, Merlen E, Robert Y, Cortet-Rudelli C, Decanter C, Jonard S (2003). Elevated serum level of anti-mullerian hormone in patients with polycystic ovary syndrome: relationship to the ovarian follicle excess and to the follicular arrest. J Clin Endocrinol Metabolism.

[35] Fallat ME, Siow Y, Marra M, Cook C, Carrillo A (1997). Müllerian-inhibiting substance in follicular fluid and serum: a comparison of patients with tubal factor infertility, polycystic ovary syndrome, and endometriosis. Fertil Steril.

[36] Laven JS, Mulders AG, Visser JA, Themmen AP, De Jong FH, Fauser BC (2004). Anti-mullerian hormone serum concentrations in normoovulatory and anovulatory women of reproductive age. J Clin Endocrinol Metabolism.

[37] Kezele PR, Nilsson EE, Skinner MK (2002). Insulin but not insulin-like growth factor-1 promotes the primordial to primary follicle transition. Mol Cell Endocrinol.

[38] Gulyas BJ, Hodgen GD, Tullner WW, Ross GT (1977). Effects of fetal or maternal hypophysectomy on endocrine organs and body weight in infant rhesus monkeys (Macaca mulatta): with particular emphasis on oogenesis. Biol Reprod.

[39] Dunaif A (1997). Insulin resistance and the polycystic ovary syndrome: mechanism and implications for pathogenesis. Endocr Rev.

[40] Knight PG, Glister C (2006). TGF-β superfamily members and ovarian follicle development. Reproduction.

