# Supplementary material for: Effect of metformin on anti-mullerian hormone levels in women with polycystic ovarian syndrome: a systematic review and meta-regression analysis of randomized controlled trials with
Source: BMC Endocr Disord. 2024 Mar 29;24:43. doi: 10.1186/s12902-024-01570-z (PMC10979616; doi:10.1186/s12902-024-01570-z)
Supplement: Supplementary file 1 — Supplementary Material 1 [file 12902_2024_1570_MOESM1_ESM.docx]

| a)   | b)   |
| --- | --- |

**Supplementary figures 1.** Sensitivity analysis of the weighted mean difference (WMD) for AMH changes in a) the single arms and b) randomized clinical trials

| a)   | b)   |
| --- | --- |

**Supplementary figures 2:** Funnel plots for evaluation of publication bias of AMH changes in a) the single arms and b) randomized clinical trials.
